# The networked micro-decision context: a new lens on transformative urban governance

**DOI:** 10.1186/s42854-023-00054-y

**Published:** 2023-04-12

**Authors:** Le Anh Nguyen Long, Rachel M. Krause, Gwen Arnold, Ryan Swanson, S. Mohsen Fatemi

**Affiliations:** 1 Department of Public Administration, University of Twente, Enschede, The Netherlands; 2 Faculty of Behavioural Management and Social Sciences, Cubicus Building, Room C-326, Drienerlolaan 5, 7522 NB Enschede, The Netherlands; 3 School of Public Affairs and Administration, University of Kansas, Lawrence, KS USA; 4 Environmental Science and Policy, University of California, Davis, CA USA

**Keywords:** Urban transformation, Decision-making, Networks, Governance, Policy learning

## Abstract

Recent large-scale societal disruptions, from the COVID-19 pandemic to intensifying wildfires and weather events, reveal the importance of transforming governance systems so they can address complex, transboundary, and rapidly evolving crises. Yet current knowledge of the decision-making dynamics that yield transformative governance remains scant. Studies typically focus on the aggregate outputs of government decisions, while overlooking their micro-level underpinnings. This is a key oversight because drivers of policy change, such as learning or competition, are prosecuted by people rather than organizations. We respond to this knowledge gap by introducing a new analytical lens for understanding policymaking, aimed at uncovering how characteristics of decision-makers and the structure of their relationships affect their likelihood of effectuating transformative policy responses. This perspective emphasizes the need for a more dynamic and relational view on urban governance in the context of transformation.

## Highlights

1. Policy-makers’ attributes and social networks both shape urban transformative capacity.

2. A city’s transformative capacity may be higher when actors involved in decision-making are more diverse.

3. Diversity spurs creativity and innovation by improving access to knowledge, learning opportunities, and skills.

4. Relationship quality, more specifically mutual trust, may help explain why some cities transform and others don’t.

5. Facilitative leadership is key in safeguarding trust and ensuring diversity among decisionmakers.

## Policy and practice recommendations

1. Invest in understanding the perspectives of key actors who make micro-level decisions on how cities respond to climate events and the relationships among these actors.
2. Facilitate transformative governance by using careful institutional design that increases diversity among decision-makers.
3. “Honest brokers” should focus on building and leveraging fungibility and trust as they pursue transformation in multi-stakeholder governance networks.

## Introduction

The increasing frequency and severity of societal disruptions diminish governments’ capacities to effectively serve their constituents. Disruptions render previous ways of operating or delivering services ineffective and threaten social well-being (Millar et al. 2018). They thereby put into focus factors which raise the vulnerability of societies, or specific societal groups. Existing policies and practices are fast becoming untenable in the face of disruptions from compounding technological, environmental, and social shocks (Phillips et al 2020; Head and Alford 2015). Governmental responses lie along a continuum, from doing nothing to engaging in transformative change that fundamentally alters a system (Moser and Ekstrom 2010; O’Brien 2012; Marshall et al. 2012; Rosenzweig and Solecki 2014).

Cascading disruptions reveal the limits of prevailing systems (Millar et al. 2018). They simultaneously highlight the need for transformative urban governance and necessitate its emergence. Transformations require social, technological, and policy innovations adopted and realized across different systemic, geographic, and temporal scales. While a variety of aims motivate calls for transformative governance, climate change and its many associated disruptions have assumed a particular urgency in driving efforts to fundamentally and intentionally alter how governing systems function.

Cities are often ground zero for climate -change-related disruption and response. They contribute as much as 70% of global greenhouse gas emissions (Boussalis et al. 2018; Hunt and Watkiss 2011) and their residents are vulnerable to many climate change-related hazards (Hobbie and Grimm 2020), both from slow-onset disasters such as drought and rapid-onset disasters like flash floods. Moreover, urban systems are typically characterized by powerful interdependencies and *tight coupling*, such that a single hazardous event can trigger cascading disruptions (e.g., infrastructure damage leading to water and power shortages) (Perrow 2000).

At the same time, cities are a vanguard of climate protection policy (Krause et al. 2021; Smeds and Acuto 2018; Watts 2017) and local control over land use, zoning, and building standards position them to develop meaningful initiatives (Einstein et al 2020; Boussalis et al 2018). New technologies, knowledge-sharing networks, and increasing wealth concentrations in urban centers likewise enable cities to act as a key locus for addressing climate change (Nguyen Long and Krause 2021; Acuto 2016). Cities are acting to protect their residents and infrastructure from sudden shocks resulting from an unpredictable and changing climate. Not all cities, however, are confronting climate change with equal vigor (Yeganeh et al. 2020; Hughes 2017). Rather than transform, some aim at preserving old systems.

Considerable effort has been put into identifying the factors that facilitate municipal climate leadership and innovation. For example, the so called ‘Lighthouse Cities’ in the European Union, which are on the cutting edge of climate and energy innovation, are typically characterized as high capacity, internationally visible cities that are enabled by their governments to lead (Eisenack and Roggero 2022). While certainly important, we argue that these types of city-level characterizations present only part of the story relevant to transformative governance. Missing from it is consideration of the backgrounds and preferences of decision-makers, as well as the nature of their interactions, which we posit are key to understanding why some cities tackle climate change despite not fitting the typical profile of a climate change leader (Homsy 2018) while others that match this profile nonetheless fail to act. In the face of this gap, there is need for scholars of urban governance to attend to the relational networks underlying urban governance decision-making and how they shape transformative capacity.

City governance unfolds as a network of networks, involving myriad interlinked and overlapping collaborations and coordination activities within and across government agencies, businesses, and community groups. We introduce the *Networked Micro-Decision Context* (NMDC) as an analytical concept through which this web of interpersonal relationships among decision-makers can be unpacked and their impact on urban transformations studied. Networks are the social structures that emerge from the presence and absence of relationships (or ties) among entities (or nodes), and from the attributes of these relationships and actors. The micro level refers to the specific decisions individuals make that affect how their city approaches climate change. Micro-level decisions are affected by and reverberate through networks, and the conjunction of these creates the context in which urban governance unfolds. The NMDC is a lens which *simultaneously accounts for how characteristics of actors involved in policy decision-making, as well as the social and relational structures enveloping them, shape urban transformation.* While its size, scope, and inclusiveness varies by issue and locale, the NMDC directs focus towards the set of actors with “a seat at the table,” who directly influence a government’s decision-making around a particular issue. Whereas a city’s elected leaders and its upper-level managers are almost always a part of the NMDC, lower level staff, representative of relevant community organizations, and private actors with interest and extensive involvement in around a particular issue may also have a seat at the table when making decisions on how to handle the disruptiveness of climate events.

An important contribution of the NMDC is its highlighting of the idea that decision-makers draw from their own personal, professional, and relational perspectives as they negotiate policy positions, share information, forge new relationships, or interrupt existing ones, in effort to shape urban governance. Although not an exclusively city-relevant concept, the NMDC has particular urban applicability. Clarence Stone’s classic work (1989) characterizes city governments as systematically lacking the capacity to effectively govern on their own, necessitating the building of long-standing partnerships with non-governmental actors. Furthermore, the confluence of actors, interests, and issues in cities makes them sites of struggle, revolution, and evolution, as documented in the various works exploring how urban actors claim to their right to the city (Lefebvre 1991; Harvey 2012).

## Transformative urban governance

The frequent occurrence of disruptions is shifting discourse around climate change governance in cities from resilience, which emphasizes a system’s ability to bounce back from a climate event (Leichenko 2011), towards transformation, emphasizing “radical, systemic change across multiple dimensions” (Hölscher and Frantzeskaki 2021: 3) that interrupts unsustainable patterns and practices (Castan Broto et al 2019: 450). In short, transformation emphasizes adaptivity. Transformation and resilience are not mutually exclusive, and efforts at achieving resiliency can be radically transformative. At the same time, in the pursuit of resiliency, old patterns and power structures may be reinforced and dimensions of sustainability neglected, such as in cases where infrastructural resilience is prioritized over the wellbeing of the urban poor (Meerow 2016; Keivani).

A city’s “transformative capacity” refers to its ability to purposefully progress towards a more sustainable state (Wolfram 2016). High levels of transformative capacity flow from “a dynamic constellation of public and private actors (able) to steer urban development in a radically different direction from historical pathways” (Castan Broto et al. 2019, 450). In other words, transformative urban governance is a networked phenomenon relying on dynamic collaboration between multiple people and organizations.

Not all cities can transform. Governance decision-making is typically incremental (Lindblom 1959; Bendor 2015; Weible 2008^b^), with past experiences substantially shaping present choices. This tendency can hamper a city’s response to disruptive events for which previous experience may offer little or flawed insight. Instead, local decision-makers must embrace *collective* and *reflexive* learning (Fedele et al. 2019; Marshall et al. 2012). Transformative decision-making requires collective “double looped” learning (Argyris 1982). In the first loop, a shared recognition that policies are failing prompts actors to seek new information and practices (Feindt 2010; Schmidt and Radaelli 2004). In the second loop, decision-makers’ experience with these newly altered practices changes their beliefs and goals for governance, leading them to generate new rules and new collective choice arrangements (Newig et al 2010; Weible 2008^a^; Howlett and Ramesh 2016). Double-looped learning concretely manifests in three types of actions: (1) information gathering (Nguyen Long and Krause 2021, Bulkeley 2006); (2) policy experimentation, during which approaches new to a jurisdiction are piloted or tested (Torrens and von Wirth 2021; Laborgne et al. 2021); and (3) using adaptive management to refine approaches based on lessons learned (Smeds and Acuto 2018).

Cities with high transformative capacity pursue forward-looking proactive policy-making(Knemeyer et al. 2009). Challenges to early recognition of disruptions may result from disruptions initially appearing minor, outside of a government’s standard scope of responsibility, or as requiring information exchange among actors not typically involved in urban governance (Hartley et al. 2019). Proactivity is predicated on recognizing the early warning signs of disruption (Wallace et al 2001) and is enhanced by inclusive and sensitive monitoring practices and mechanisms to integrate relevant findings into ongoing policy discussions (Rosenzweig and Solecki 2018; Wolfram 2016).

System change involves greater short-term risk than strategies focused on coping or otherwise “muddling through” (Lindblom 1959). These risks can be both political and financial. For example, elected officials who attempt to fundamentally change the status quo via financially burdensome policy instruments such as taxes and fines risk electoral rejection from voters (Harrison 2012; Slack 2012). Redistributive policies that reallocate benefits once reserved for advantaged groups are also likely to be controversial and risky (Lowi 1972; Hays 1990). Transformative change often requires officials to make difficult decisions and commit to bearing costs in the present to achieve uncertain future gains (Fedele et al. 2019; Sarewitz et al. 2003).

Even in the absence of disruption, the manifold issues, stakeholders, and uncertainties commonly implicated when operating in urban spaces (Harvey 2012) combined with the complexity inherent to being situated in a nested, multi-level policymaking environment often give rise to transformation-triggering challenges. In today’s polycentric urban systems, an array of governance styles including networked governance (e.g., Provan and Milward 2001; Sørensen and Torfing 2009), collaborative governance (e.g., Ansell and Gash 2007; Emerson et al. 2012) and adaptive governance (e.g., Folke et al. 2005; Adger 2009) can be observed. While distinct, they all emphasize a *participatory turn* in urban governance and collaboration between and across diverse government agencies and non-governmental actors. This understanding of governance contrasts sharply with the conventional view of government as siloed, hierarchical, and hampered by red tape, and coheres with our claim that a greater understanding of the networked nature of city governance will uncover new insights into urban transformations.

Although the previously identified approaches recognize the decentralized and participatory nature of urban governance, the NMDC lens advances this further by highlighting the identities of involved actors and the evolving nature of their relationships in the achievement of transformative capacity. Its micro-level, relational focus differs from much of the extant literature on local policy making, which often treats cities as singular entities whose decisions are explained by a combination of citizen preferences, institutional characteristics, and governmental capacity (Tausanovitch and Warshaw 2014; Jimenez 2020). A partial exception is research on policy entrepreneurs, change agents often critical to the uptake of transformative policy innovations (Mintrom 1997; Mintrom and Vergari 1998). While this scholarship examines the influence of specific players in city government, such as managers and mayors (e.g., Yi and Chen 2019; Dzigbede et al 2020), few studies fully and simultaneously account for the range of individuals whose resources, knowledge, and behaviors shape interactions and the policies that emerge from them. The NMDC responds to this oversight.

## Using the NMDC to open the policymaking black box

The NMDC zooms out from the individual and in from the city level. Governance scholarship largely relies on decision models focused on individual cognition, preferences, and choices. Policy participants are understood as people who adhere to a logic of appropriateness or consequences (March and Olsen 1983), satisficers who choose the first acceptable option (Simon 1947), incremental learners whose decision-making rarely branches far from the root of institutional experience and knowledge (Lindblom 1959; Weible 2008^b^), or loss-adverse actors who prefer options that do the least damage rather than have the potential to yield the most gain (Tversky and Kahneman 1991). To varying degrees, these perspectives also deemphasize or ignore that decision-makers are embedded in social structures, where they can influence and be influenced by others and often make decisions collectively. Understanding these decisions require understanding relational and power dynamics. Previous perspectives were largely devised to explain decision-making during periods of relative stability, but understanding transformative governance requires a more dynamic and relational view on decision-making (Hartley et al. 2019; Howlett and Ramesh 2016). The NMDC offers an analytical framework to help researchers fill this gap.

Similar to networked governance theories, the NMDC framework emphasizes resource dependencies that stimulate exchange relationships among participants who can offer needed material, knowledge, and social resources. Aligning with the idea that there can be diverse pathways to transformation (Rosenzweig and Solecki 2018; Hölscher and Frantzeskaki 2021), the NMDC concept aides the comparison of an array of governance configurations. It further emphasizes social identities and the role that key actors play in guiding and managing relationships. Building on this, we offer three propositions about how individual and relational characteristics, as highlighted by the NMDC, shape a city’s transformative potential.

### Postulate 1: Greater professional diversity can encourage learning-oriented and proactive governance across a network of decision-makers

The NMDC highlights the plurality of decision-makers in urban governance, and the value of heterogeneity among them. Rarely is a policy choice made by a single person, and group diversity, in general, has been found to improve team performance, particularly in areas of innovation and creativity (Bell et al. 2011; Weigand 2007; Barkema and Oleg 2007), and can substantially improve group cognition and ability to address non-routine problems (Loyd et al. 2013; Phillips et al. 2014). Although the many types of diversity can each affect group performance differently (Page 2019), the NMDC guides our focus toward professional diversity. The range of professional backgrounds and responsibilities across members of a decision-making network affects the extent to which they share language, norms, and communication and problem-solving styles (Bell et al. 2011). Although it may decrease efficiency, working with individuals from various professional backgrounds often requires otherwise taken for granted choices to be explained or justified, causing a switch from fast-thinking to slow-thinking. In this slow-thinking mode, decision-making is improved as more variables and alternatives are considered (Kahneman 2012).

Additionally, a professionally diverse group is more likely connected to a broader range of information channels, which together are more likely to contain a signal of coming disruption (c.f. Hon and Brunner 2000) laying the groundwork for the proactivity which is a hallmark of transformative governance. The resulting idea-rich environment can foster learning and generate new insights about how to make policy proactively. Cognitive diversity raises the likelihood that different ideas will be applied to a problem (Barkema and Oleg 2007), as exposure to other ways of working and thinking shifts mindsets and beliefs.

Organizational diversity facilitates learning by giving network participants a wealth of examples of different approaches, materials, authority, and informational and legitimacy resources. An urban governance network comprised of diverse units can creatively borrow rules, norms, and strategies from one another, and combine them to tackle novel dilemmas (Merrey and Cook 2012). Conflict is one oft-noted disadvantage of group-level diversity (Pelled et al. 1999). For example, in some US cities, attempts to pursue sustainability improvements have been hindered by a tension between the planners and engineers on city staff, who tend to approach the issue from different perspectives (Krause and Hawkins 2021). Diversity facilitates the emergence of faultlines around which competing clusters form and compete (Lau and Murnighan 1998). Previous research has shown that in the face of competing demands, local politicians are less likely to adopt a policy innovation, ergo they are less likely to act transformative-ly. Under certain conditions, however, competition may be healthy for policy innovation, especially when subgroups develop different solutions contemporaneously (c.f. Ostrom 2012) and learn from each other in an effort to stay ahead (Rampersad et al. 2010).

Diversity is the result of choices made by network members. As governance participants repeatedly interact, over time and in different contexts, they begin to prefer some contacts over others (Newig et al. 2018). The composition of the resulting network can range from being very homogenous, e.g., restricted to a few public officials, to very diverse, involving scientists, representatives from industry and civil society, diverse government agencies, and highly engaged citizens from all walks of life. It bears mentioning that although governance fora are often biased towards the participation of public officials, industry representatives, or wealthy citizens (Cardullo and Kitchin 2019; Irvin and Stansbury 2004), broad citizen participation can be enhanced and supported through careful consideration and design of the participatory process (Fung 2006; Torfing et al. 2019). For instance, relaxing strict procedural rules can facilitate policy co-design with citizens (Callens 2023). The more diverse an NMDC is, the more attention must be paid to institutional design (Ansell and Gash 2007).

### Postulate 2: Mutual trust among network participants can increase overall proactivity and willingness to take risk

The NMDC places focus not just on *who* participates, but also on the quality of their relationships. We posit that trusting relationships are fundamental drivers of transformation. Trust encourages knowledge exchange by decreasing the transaction costs and risks related to sharing sensitive or preliminary knowledge. Trust encourages team members to own up to mistakes, helping an organization avert even larger crises (Cho and Poister 2013; Costigan et al.^2006^). The wish to maintain trust also encourages partners to treat knowledge and resources sharing carefully. Essentially, trust lessens the burden of managing a group of autonomous decision-makers and is the lynchpin for self-organization. Trusting relationships enable the network to operate outside or at the margins of conventional practices, activities often necessary for pursuing transformative change (Lomnitz and Sheinbaum 2004). While the impulse for actors operating in uncertain environments is to fall back on standard rules and formal procedures (Zhou 1997), a decision-making network may be better equipped to pursue transformative governance when it is structurally flexible and comprised of participants willing and able to respond to new exigencies by adjusting the rules and protocols. For example, actors are more likely to pursue risky experiments if they are confident that others in the network will support them, rather than blame them or seek competitive advantage, should attempts go awry (Fawcett et al. 2012).

Moreover, mutual trust is needed for network participants to draw benefits from structural flexibility and fungibility. Structural flexibility is closely tied with network fungibility. In fungible networks, participants, be they individuals or organizations, can assist or step in for each other if one participant is spread thin by disruption-related challenges (Lipton 1984; Russ-Eft 2000; Berkes et al. 2003; Low et al. 2003; Walker et al. 2006). If network participants know that a network partner is willing and able to temporarily take on their functions if problems arise, all have greater confidence that the governance system will continue reliably serving the public. By providing a safety net that mitigates risks turning into losses, network fungibility lowers the cost of risky behavior and facilitates learning and policy experimentation (Smeds and Acuto 2018). As such, fungibility can encourage transformative governance by reducing the adverse consequences of failure (Berkes et al. 2003; Low et al. 2003; Walker et al. 2006).

However, trust within the network cannot be taken for granted, even when it is composed of rather stable relationships. For instance, public officials' lack of trust in citizens’ competencies can hinder collaboration (Wagenaar 2007). On the flipside, if past conflicts among network members fosters distrust towards powerful^1^ members (like government officials), communication and cooperation can degrade, with potentially devastating consequences for policymaking. In both cases, the literature suggests that face-to-face communication and repeated information sharing is key to increasing trust, transparency and accountability (Newig et al. 2018).

### Postulate 3: Facilitative leadership may enable a network to leverage diversity and build trust.

There are many different types of leaders: They can be inspirational, transactional, or transformative (Sancino et al. 2021) and fulfill different roles, from sponsoring, to championing, to facilitating (Crosby and Bryson 2018). Of these, facilitative leaders, who are known for being impartial and fair arbitrators (Ansell and Gash 2007), are crucial for building transformative capacity in urban governance. This reputation grants them the legitimacy to set and enforce rules of engagement, resolve conflict, and explore mutual gains (Newig, et al. 2018). They work to create an environment where ideas can be shared openly and integrated creatively, to stimulate new understandings of a challenge and its solutions.

Facilitative leaders encourage inclusivity and enable meaningful participation (Lasker 2003). The opportunity to meaningfully express voice is a first step towards having power. Thus, facilitative leaders open the door to broader involvement in deliberations about “who gets what, when, and how” from the policy process (Lasswell 1936). Disadvantaged citizens like the urban poor may not be able to participate as effectively in the network as other stakeholders. Moreover, powerful actors may see transformations as a threat, since transitions can lead to new distributions of power in society, and may deploy their resources and influence to frustrate them. Indeed, “radical changes” are not always positive (Torrens and von Wirth 2021) or positive for all groups (Elmqvist et al. 2019; Hölscher and Frantzeskaki 2021). Facilitative leaders who successfully advance transformation recognize power disparities and intentionally seek to mitigate these dynamics.

A leader acting as an “honest broker” can help mediate unhealthy power dynamics in a network. For example, in a case study of the post-disaster reconstruction of a lower-income neighborhood in the Netherlands, Denters and Klok (2010) observe that broad-based participation by residents, public officials, and scientific experts was enabled through the engagement of an independent “process facilitator” who chaired and moderated meetings and ensured the representation of affected participants. The decision-making networks best-equipped to pursue transformative governance may share and shift leadership among members when confronting different challenges, helping ensure that leadership is prosecuted by someone who can remain “above the fray.”

## Conclusion: Building transformative capacity from within

Societies worldwide are facing threats from a range of looming disruptions, including climate change, severe weather events, cybersecurity threats, and pandemics. We urgently need to understand how governments can transform practices and policies to improve their capacity to deal with such disruptions. As an innovative analytical lens, the *Networked Micro-Decision Context* advances this aim. The NMDC examines the micro-level dynamics within the policymaking black box, focusing on individuals and organizations centrally engaged in governance, their characteristics, and their relational dynamics. By observing patterned interactions among core members of an urban governance network and asking how well these interactions foster key preconditions for transformative governance—learning proactivity, and willingness to invest in risky policy making—we offer an approach for quantifying and assessing transformative urban governance. In so doing, we help move the construct of *transformativeness* from metaphor to a measure useful to decision-makers and scholars alike.

The NMDC draws focus to (1) the collective aspect of decision-making, and how diversity contributes to this, (2) the quality of relationships among decision-makers, specifically extent of trust, and (3) the role of key actors in and how their leadership style can promote or discourage transformative governance. We are not alone in calling attention to these attributes (e.g. Crosby and Bryson 2018; Ansell and Gash 2007; Fung 2006), but to our knowledge we are the first to consider them simultaneously while linking them to characteristics (learning, proactivity, and risk acceptance) that shape a city’s transformative capacity. The next step is empirically testing the postulates we advance above as well as the utility of the NMDC lens in helping understand when, why, and to what extent transformative governance occurs in urban contexts. To that end, a range of analytical approaches can be employed. For example, social network analysis could be used to quantify and compare network structures and composition across cases, assessing whether structural flexibility appears to promote mutual learning (Postulate 2). Qualitative interviews, case studies, and ethnographies can be used to dig interpersonal dynamics within a network and assess whether and when greater professional diversity spurs unproductive conflict versus productive competition in disruption-related problem-solving (Postulate 1). Statistical analysis could help evaluate whether, greater professional diversity within a network correlates with its members pursuing risky policies to tackle disruptions, such as adopting costly coastal community relocation programs in the face of sea level rise (Postulate 2) or whether a network that appears to feature a facilitative leader are more smoothen the rollout of pilot or demonstration projects or other hallmarks of policy learning (Postulate 3). Indeed, empirical analysis will help assess the degree to which learning orientation, policy proactivity, and risk-acceptance in policymaking are in fact hallmarks of transformative governance, and whether there are other or additional dimensions for which we should account. We look forward to this important conversation between theory and empirics—while keeping in mind the ultimate goal of helping practitioners and their communities emerge from disruptive experiences with greater resiliency and improved quality of life.

## Data Availability

No data was used to develop this paper.

## Abbreviation

NMDC: Networked Micro-Decision Context

## Footnotes

1 Power can have many faces (Lukes 2005) and that different actors may be endowed with different levels of each face. We further expect that transformation can spark shifts in power (Avelino and Wittmayer) which may in turn impact NMDC dynamics.
